# Cellular metabolic response to DNA damage

**DOI:** 10.1186/1753-6561-6-S3-P24

**Published:** 2012-06-01

**Authors:** Seung Min Jeong, Marcia C Haigis

**Affiliations:** 1 Department of Cell Biology, The Paul F. Glenn Labs for the Biological Mechanisms of Aging, Harvard Medical School, Boston, MA USA

## 

DNA damage elicits a cellular signaling response that initiates cell cycle arrest and DNA repair. The metabolic response to DNA damage is largely unknown. Here we report a novel metabolic response to genotoxic stress. DNA damage triggers a critical block in the uptake of glutamine, a mitochondrial substrate essential for cellular proliferation. Sirtuins regulate both cellular metabolism and stress responses. We found mitochondrial SIRT4 is involved in the metabolic response to DNA damage. These results suggest that the metabolic adaptation is important for cellular DNA damage response.

